# The Loss of α- and β-Tubulin Proteins Are a Pathological Hallmark of Chronic Alcohol Consumption and Natural Brain Ageing

**DOI:** 10.3390/brainsci8090175

**Published:** 2018-09-11

**Authors:** Wajana L. Labisso, Ana-Caroline Raulin, Lucky L. Nwidu, Artur Kocon, Declan Wayne, Amaia M. Erdozain, Benito Morentin, Daniela Schwendener, George Allen, Jack Enticott, Henry K. Gerdes, Laura Johnson, John Grzeskowiak, Fryni Drizou, Rebecca Tarbox, Natalia A. Osna, Kusum K. Kharbanda, Luis F. Callado, Wayne G. Carter

**Affiliations:** 1 School of Medicine, University of Nottingham, Royal Derby Hospital Centre, Derby DE22 3DT, UK; wajana.lako@aau.edu.et (W.L.L.); A.Raulin@sussex.ac.uk (A.-C.R.); lucky.nwidu@uniport.edu.ng (L.L.N.); msxamk@nottingham.ac.uk (A.K.); stxdw5@exmail.nottingham.ac.uk (D.W.); amaia.m.erdozain@gmail.com (A.M.E.); danielaschwendener@gmail.com (D.S.); george.allen@doctors.org.uk (G.A.); jack.enticott@btinternet.com (J.E.); henry.k.gerdes@gmail.com (H.K.G.); laurajohnsonLJ@hotmail.co.uk (L.J.); stxjwg@exmail.nottingham.ac.uk (J.G.); sbafd@exmail.nottingham.ac.uk (F.D.); mdzrr@exmail.nottingham.ac.uk (R.T.); 2 School of Medicine, Addis Ababa University, Addis Ababa 1000, Ethiopia; 3 École nationale supérieure de chimie de Montpellier, 34090 Montpellier, France; 4 Department of Experimental Pharmacology and Toxicology, University of Port Harcourt, Port Harcourt 500262, Rivers State, Nigeria; 5 Department of Pharmacology, University of the Basque Country, Leioa-Erandio 48940, Spain; lf.callado@ehu.eus; 6 Centro de Investigación Biomédica en Red de Salud Mental, Madrid 28029, Spain; 7 Section of Forensic Pathology, Basque Institute of Legal Medicine, Bilbao 48001, Spain; morentin.b@justizia.eus; 8 Research Service, Veterans Affairs Nebraska-Western Iowa Health Care System, Omaha, NE 68105, USA; nosna@unmc.edu (N.A.O.); kkharbanda@unmc.edu (K.K.K.); 9 Departments of Internal Medicine and Biochemistry & Molecular Biology, University of Nebraska Medical Center, Omaha, NE 68105, USA

**Keywords:** acetylation, ageing, alcoholism, alcohol-related brain damage, α-tubulin, β-tubulin, HDAC6, MAP-2, MAP-tau, pre-frontal cortex

## Abstract

Repetitive excessive alcohol intoxication leads to neuronal damage and brain shrinkage. We examined cytoskeletal protein expression in human *post-mortem* tissue from Brodmann’s area 9 of the prefrontal cortex (PFC). Brain samples from 44 individuals were divided into equal groups of 11 control, 11 alcoholic, 11 non-alcoholic suicides, and 11 suicide alcoholics matched for age, sex, and *post-mortem* delay. Tissue from alcoholic cohorts displayed significantly reduced expression of α- and β-tubulins, and increased levels of acetylated α-tubulin. Protein levels of histone deacetylase-6 (HDAC6), and the microtubule-associated proteins MAP-2 and MAP-tau were reduced in alcoholic cohorts, although for MAPs this was not significant. Tubulin gene expressions increased in alcoholic cohorts but not significantly. Brains from rats administered alcohol for 4 weeks also displayed significantly reduced tubulin protein levels and increased α-tubulin acetylation. PFC tissue from control subjects had reduced tubulin protein expression that was most notable from the sixth to the eighth decade of life. Collectively, loss of neuronal tubulin proteins are a hallmark of both chronic alcohol consumption and natural brain ageing. The reduction of cytosolic tubulin proteins could contribute to the brain volumetric losses reported for alcoholic patients and the elderly.

## 1. Introduction

Imbibing moderate quantities of alcohol is commonplace within the social culture of many countries. However, alcohol use disorders are also prevalent within many developed countries [1]; with excessive and harmful alcohol usage responsible for approximately 1 in 7 deaths in men (≈14%) and 1 in 13 deaths in women (≈8%) within the European Union [2], and ≈6% of all deaths worldwide per year [3].

Although alcohol consumption damages tissues and organs including the brain, the neuro-toxicological properties of alcohol are incompletely understood. Alcoholics experience undesired emotional and behavioral problems [4,5], and are approximately twice as likely to suffer from a major depressive episode compared to those that are not alcohol-dependent [6]. In order to develop new modalities for the treatment of alcoholism, an improved understanding of the molecular neurobiology and neuropathology of alcohol is required [7,8,9,10,11].

The ability of chronic alcohol abuse to trigger global neuronal cell death has been a topic of debate, with neurotoxicity of alcohol likely cell-type specific, and display brain regional bias [11,12,13,14,15,16]. Pathological analyses, as well as structural neuroimaging studies, have reported brain volumetric losses in alcoholic cohorts, with reduced cortical and subcortical grey and white matter shown [12,13,14,15,16,17,18,19,20,21,22,23,24,25,26,27]. The frontal regions of the cortex have been recognized as particularly susceptible to the damaging effects of alcohol [12,15,16,17,18,19,20,21,22,23,24,25,26,27,28,29]. Reduced brain cortical thickness in alcoholics has also been attributed to comorbidity factors such as smoking, illicit drug use, and a natural decline in size associated with ageing [17,19,28].

Alcohol-induced neuronal loss may in part be balanced by periods of neurogenesis during abstinence [30] with a partial reversion of the volumetric losses detected using neuroimaging [19,21,27,28,31,32].

The prefrontal cortex (PFC) is involved in a number of higher-order (executive) functions and receives multimodal input via the visual, auditory, and somatosensory cortex [33]. Hence, alcohol-related damage to the PFC and dorsolateral mesolimbic system may underpin some of the emotional and behavioral abnormalities exhibited by alcoholics, including reward-seeking behavior.

The common comorbidity of alcoholism with other neuropsychiatric disorders led us to investigate whether there was overlapping molecular damage to the PFC in alcoholic patients, and alcoholics that had committed suicide, when compared to their corresponding age- and sex-matched controls (Demographic Table 1 and Table 2). From both mRNA and proteomic studies, regional and heterogeneous reductions of microtubule (MT) and microtubule-associated protein (MAP) expressions have also been reported within the mammalian brain in response to alcohol exposures [10,34,35]. To extend these studies, we have focused upon expression of the α- and β-tubulin proteins and MAPs in human alcoholic and suicide alcoholic cohorts. Additionally, we quantified expression levels of brain tubulin proteins and MAPs after administration of alcohol for 4 weeks to laboratory rats. This enabled us to assess whether protein changes observed in human alcoholics could be modelled after chronic alcohol exposure to rats. Finally, we determined the relationship between the protein expression of human brain tubulins and subject age (Demographic Table 3).

## 2. Materials and Methods

### 2.1. Human Brain Samples and Ethics Statement

The Neuropsychopharmacology Research Group at the Department of Pharmacology of the University of the Basque Country (UPV/EHU) supplied the human *post-mortem* samples used in these studies (https://www.ehu.eus/en/web/neuropsicofarmacologia/aurkezpena). Brain collection is registered at the National Biobank Register of the Spanish Health Department with the number C.0000035 (https://biobancos.isciii.es/ListadoColecciones.aspx).

Human brains were obtained at autopsy from 11 control subjects with no *ante-mortem* neurological or psychiatric disorders, 11 non-suicidal alcoholic subjects who died of natural causes, 11 non-alcoholic suicide subjects that had committed suicide and presented psychiatric diagnoses other than alcoholism, and 11 suicidal alcoholic subjects that had committed suicide without any other additional psychiatric diagnoses [36] (Demographic Table 1 and Table 2). The collection of brains complied with the policies of research and ethical review boards for *post-mortem* brain studies (Basque Institute of Legal Medicine, Bilbao). Spanish legislation at the time of sample collection did not require written informed consent from the next of kin for use of these *post-mortem* samples in research. Furthermore, the United States Department of Health and Health Services (DHHS), and Food and Drug Administration (FDA) regulations do not define *post-mortem* brain specimen analyses as human research.

The diagnosis of alcoholism was carried out according to the Diagnostic and Statistical Manual of Mental Disorders (DSM-III-R, DSM-IV, or DSM-IV-TR; American Psychiatric Association) or International Classification of Diseases criteria (ICD-10; World Health Organization). Clinicians in charge of the patients established all diagnoses prior to death. Eleven sets of control, alcoholic, suicide, and suicide alcoholic subjects were matched for gender, age, and *post-mortem* delay. For each study participant, blood toxicological screening for alcohol and psychotropic drugs was undertaken. Table 1 and Table 2 are a summary of the demographic characteristics of the subjects included in this part of the study. Samples from the prefrontal cortex (Brodmann’s area 9) (BA 9) were macroscopically dissected at the time of autopsy and immediately stored at −70 °C until required.

Table 3 summarizes the demographic characteristics of the control subjects used for the ageing brain study.

### 2.2. Rats

Studies were undertaken with four pairs of male Wistar rats (180–200 g body weight) fed Lieber DeCarli control and ethanol liquid diets for 4 weeks [37]. After sacrifice, the brains from rats were rapidly removed, flash-frozen, and then stored at −80 °C until required. The Institutional Animal Care and Use Committee at the Omaha Veterans Affairs Medical Center, Nebraska, USA approved the care, use, and procedures performed on these rats. Rat brains were transported on dry ice to the University of Nottingham, UK, for processing.

### 2.3. Brain Tissue Homogenization

Human brain tissue from Brodmann’s area 9 (typically ≈100 mg/individual sample) or rat whole brain were homogenized on ice using a glass hand-held homogenizer in 10 volumes of buffer (20 mM Tris/HCl pH 7.4, 1 mM ethylenediaminetetraacetic acid (EDTA), containing one tablet of protease inhibitor cocktail (Roche), and 1:100 dilution of phosphatase inhibitor cocktail (Sigma-Aldrich, Dorset, UK). Homogenates were centrifuged at 500× *g* for 10 min at 4 °C to pellet the nuclear fraction and cell debris. The supernatant was then centrifuged at 21,100× *g* for 40 min at 4 °C to produce a crude cytosolic preparation. The pellet from this centrifugation constituted the plasma membrane-enriched fraction and was resuspended in ≈10 volumes of the homogenization buffer. Similarly, the nuclear pellet was resuspended in ≈10 volumes of homogenization buffer. Nuclear, cytosolic, and membrane-enriched fractions were flash-frozen and stored at −80 °C until required. Protein concentration in each fraction was determined by a modified Lowry assay (Bio-Rad, Hertfordshire, UK) using a bovine serum albumin standard set (Bio-Rad 500-0207) as protein standards.

### 2.4. One-Dimensional Polyacrylamide Gel Electrophoresis (1-D PAGE)

Human prefrontal cortex fractions or rat whole brain homogenates were separated on 10% Bis-Tris NuPAGE Novex pre-cast gels (Fisher Scientific, Loughborough, UK) (typically 20 µg/gel lane) run with 3-morpholinopropane-1-sulfonic acid (MOPS) running buffer according to published procedures [10]. Resolved proteins were either stained with colloidal Coomassie blue and then densitometry performed using an Odyssey laser scanner (LI-COR Biosciences, Lincoln, Nebraska, USA), or proteins electroblotted at 80 V for 2 h to a polyvinylidene difluoride (PVDF) (Millipore, Burlington, MA, USA) membrane for Western blotting. Protein homogenates were typically resolved 3–4 times by gel electrophoresis for each brain sample, with representative images used for Figures. Similarly, protein extracts were resolved 3–4 times for Western blotting analysis, with representative images used for Figure production, and protein level quantitation.

### 2.5. Western (Immuno) Blotting

PVDF membranes were prepared according to previously published procedures [38,39]. Membranes were incubated overnight at 4 °C with the primary antibody of interest in a blocking buffer. The antibodies used in this study were directed against the following proteins, and at the dilutions specified: rabbit polyclonal to human HDAC6 (Santa Cruz, TX, USA, sc-11420) at 1:1000; mouse monoclonal to α-tubulin (Santa Cruz, TX, USA, sc-8035) at 1:1000; mouse monoclonal to acetylated α-tubulin (Santa Cruz, TX, USA, sc-23950) at 1:1000; rabbit polyclonal to β-tubulin (Santa Cruz, TX, USA, sc-9104) at 1:1000; rabbit polyclonal to MAP-2 (Santa Cruz, TX, USA, sc-20172) at 1:1000; goat polyclonal to MAP-Tau (Santa Cruz, TX, USA, sc-1995) at 1:1000; and mouse monoclonal antibody to glyceraldehyde 3-phosphate dehydrogenase (GAPDH) (Abcam, Cambridge, UK, ab8245) at 1:3000 as a gel loading control for cytosols. Blots were washed in phosphate-buffered saline containing 0.05% Tween-20 (PBS-T) and then incubated for 1 h at room temperature with their corresponding horseradish peroxidase conjugated anti-species secondary antibody (polyclonal goat anti-rabbit, goat anti-mouse, or goat-anti rabbit immunoglobulins, purchased from Dako (Cheshire, UK), (P0448, P0447, and P0449 respectively) at 1:2000 dilution in blocking buffer. Antibody immunoreactivity was visualised using SuperSignal West Pico Chemiluminescent substrate (Pierce, Loughborough, UK) or Clarity Western ECL Substrate (Bio-Rad, Hertfordshire, UK) with the light generated captured using a Chemi Doc device (Bio-Rad, Hertfordshire, UK) or Chemi Doc MP imaging system (Bio-Rad, Hertfordshire, UK), respectively. Blots were quantified using the image processing and analysis program, Image J (version 1.51, NIH, USA, https://imagej.nih.gov/ij/), developed at the National Institutes of Health, or for the most part using the intrinsic software (Image Lab 5.0, Bio-Rad, Hertfordshire, UK) of the Chemi Doc MP imager. Image Lab 5.0 software was set at auto-exposure to generate a signal across the blot that was both linear and that did not reach signal saturation. Western blot quantitation was normalized to either GAPDH values and/or total lane protein. The mean signal density for quantitation of control samples was set at 100% of the protein signal.

### 2.6. Quantitative Real-Time Polymerase Chain Reaction (qRT-PCR)

For RNA production, typically 50–100 mg tissue/individual sample was homogenized using a Fast Prep-24 tissue grinder (MP Biomedicals, Loughborough, UK), and RNA extracted using an All Prep Mini-Kit (Qiagen, Manchester, UK) according to the manufacturer’s protocol. Equal amounts of RNA extracted from samples were reverse transcribed and used as a template for qRT-PCR using a Gene Amp PCR system 9700 according to the manufacturer’s guidelines. Quantitative RT-PCR was undertaken using a CFX Connect Real-Time system (Bio-Rad, Hertfordshire, UK). Probes for α-tubulin isoform 1A (TUBA1A, product 4331182) and β-tubulin isoform 2B (TUBB2B, product 4351372) were purchased from Life Technologies (Loughborough, UK). Beta actin (ACTB), beta-2-microglobulin (B2M), and tyrosine 3-monooxygenase/tryptophan 5-monooxygenase activation protein zeta (YWHAZ) were assayed independently under similar conditions were used as housekeeping genes. The means of the three reference genes provided target normalization. The normalized cycles to threshold (∆Ct) values for each sample were calculated. Figures display the relative gene expression change to those of the control tissue samples.

### 2.7. Statistics

For quantitation of protein bands on Western blots, or gene expression changes for qRT-PCR, statistical comparison between data means was performed with either an ANOVA with Dunnett’s multiple comparison test or a Student’s *t*-test using GraphPad Prism software (version 7.03, GraphPad Software Inc., San Diego, CA, USA), with results expressed graphically as means ± SEMs. Differences between protein expression and patient age was determined using a non-parametric ANOVA (Kruskal-Wallis test). A Spearman rank-order correlation coefficient was used to assess the relationship between age or sex and protein expression. Statistical significance was set at a *p* value of <0.05.

## 3. Results

### 3.1. Reduced α- and β-Tubulins and Microtubule-Associated Proteins in the PFC of Alcoholic Subjects

**Figure 1:**
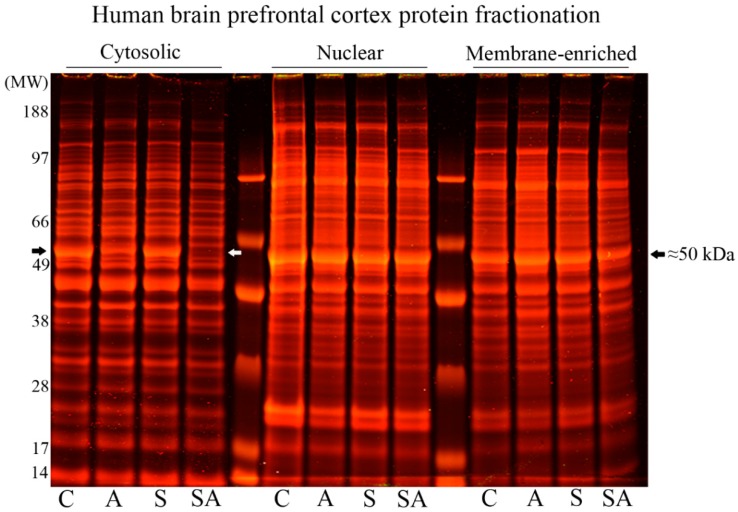
Resolution of human PFC proteins from cytosolic, nuclear, and membrane-enriched fractions from control (C), alcoholic (A), suicide (S), and suicide alcoholic (SA) patients. Prefrontal cortex proteins were resolved by one dimensional polyacrylamide gel electrophoresis and then stained with colloidal Coomassie. Reduced protein staining at ≈50 kDa (marked with arrowheads) was solely in the cytosolic protein fractions of alcoholic and suicide alcoholic patients.

**Figure 2:**
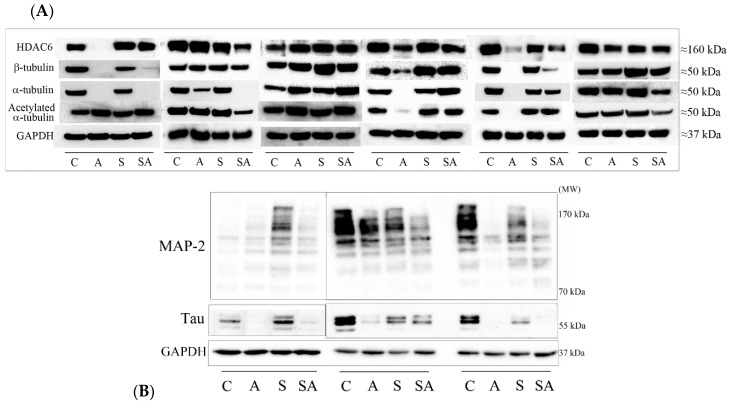
Protein levels of PFC proteins visualized by Western blotting. (**A**) Cytosolic proteins resolved by one dimensional polyacrylamide gel electrophoresis (1D PAGE) were Western blotted using antibodies to HDAC6, β-tubulin, α-tubulin, acetylated α-tubulin, and GAPDH. Blotting panels from six sets of matched control (C), alcoholic (A), suicide (S), and suicide alcoholic (SA) patients are included. (**B**) Cytosolic proteins resolved by 1D PAGE were Western blotted with antibodies to MAP-2, MAP-tau, and GAPDH. Blotting panels from three sample sets of matched control (C), alcoholic (A), suicide (S), and suicide alcoholic (SA) patients are included.

We examined PFC tissue from 11 sets of control, alcoholic, suicide, and suicide alcoholic subjects (Demographic Table 1 and Table 2). PFC tissue was homogenized and resolved by differential centrifugation to produce crude nuclear, cytosolic, and membrane-enriched fractions. Proteins were then resolved by one-dimensional polyacrylamide gel electrophoresis. Protein expressions within nuclear and membrane-enriched fractions were visually similar for all cohorts, but cytosolic levels of an ≈50 kDa protein were reduced only in alcoholic and suicide alcoholic cohorts (Figure 1). In accordance with previous studies [10], we confirmed that this major protein loss was attributed to reduced α- and β-tubulins via Western blotting (Figure 2A), and from mass spectrometry (refer to Supplementary Data).

**Figure 3:**
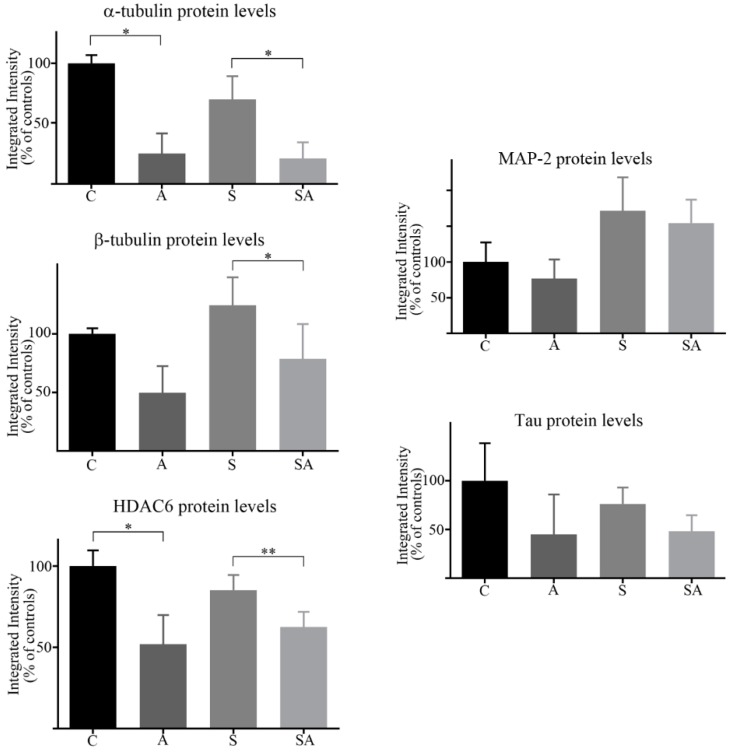
Quantitation of Western blots. Western blots of proteins from control (C), alcoholic (A), suicide (S), and suicide alcoholic (SA) patients were quantified using densitometry, with GAPDH levels used for blot normalization. Histograms are representative of means ± standard deviation, *n* = 9–11. For marked significance: * = *p* < 0.05, ** = *p* < 0.01. Control values were set at 100%.

A number of proteins bind and interact with microtubules including histone deacetylase 6 (HDAC6), the major α-tubulin deacetylase, and the microtubule associated proteins (MAPs): MAP-2, and MAP-tau. We therefore investigated whether their protein expressions were also reduced in the cytosolic fractions from alcoholic cohorts. Although all three proteins were reduced in level in alcoholic cohorts, only the reduction of HDAC6 reached significance (Figure 2A,B and Figure 3). We also observed an increased acetylation of α-tubulin in both alcoholic cohorts (Figure 2A), consistent with our previous publication [10].

Western blot quantitation (Figure 3) revealed a significant 75% (*p* = 0.03) reduction in α-tubulin in alcoholic subjects relative to controls, and a significant 70% decrease (*p* = 0.049) in α-tubulin in suicide alcoholic subjects relative to non-alcoholic suicide patients. The ≈30% reduction of α-tubulin observed for suicide subjects relative to controls was not significant. Reductions in β-tubulin of 50% in alcoholics compared to controls were non-significant due to extensive sample variability, but there was a significant 37% reduction (*p* = 0.016) of β-tubulin in suicide alcoholic patients compared to non-alcoholic suicide cohorts.

HDAC6 protein levels were significantly reduced 48% in alcoholic subjects (*p* = 0.021) compared with controls, and 27% (*p* = 0.003) in suicide alcoholics relative to non-alcoholic suicide subjects. HDAC6 expression was 15% lower in suicide subjects relative to controls, but this was not significant (Figure 3).

Western blots of MAP-2 and MAP-tau typically detected the presence of eight and three major protein bands, respectively, for which total immune-reactivity was quantified in both cases (Figure 2B and Figure 3). There was considerable variability between MAP-2 and MAP-tau protein expression between individuals. Although both alcoholics and suicide alcoholics had 23% and 10% reduced total expression of MAP-2, respectively, compared with the control and suicide counterparts, neither reduction reached significance. Similarly, reduced MAP-tau protein levels for alcoholic cohorts were not significant.

### 3.2. α- and β-Tubulin Gene Expression Were Moderately Increased in Alcoholic Cohorts

**Figure 4:**
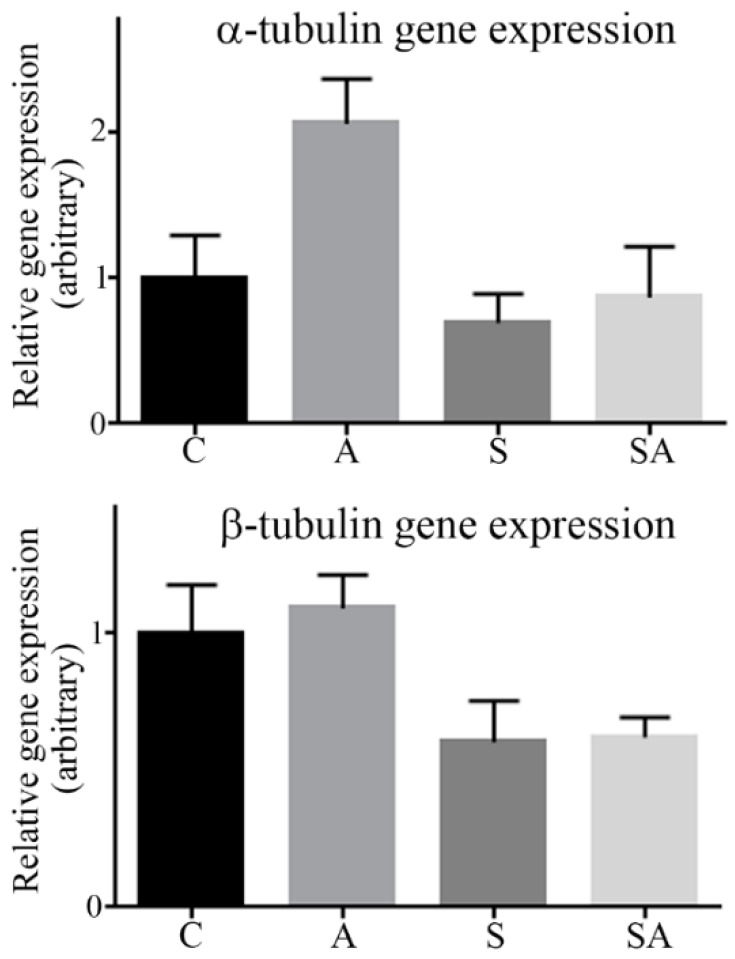
Quantitation of α- and β-tubulin gene expression by qRT-PCR. Relative gene expression of α-tubulin and β-tubulin in control (C), alcoholic (A), suicide (S), and suicide alcoholic (SA) patients was quantified using qRT-PCR. Expression of the genes beta actin, beta-2-microglobulin, and tyrosine 3-monooxygenase/tryptophan 5-monooxygenase activation protein zeta were used as housekeeping genes. Histograms are representative of means ± SDs, *n* = 6. Control values were set at 1.

Reduced α- and β-tubulin protein expression observed for alcoholic cohorts could have arisen from reduced transcription. This was assessed using qRT-PCR with probes specific for tubulins. Contrary to protein levels, there was an ≈2-fold (but just non-significant (*p* = 0.06)) increase of α-tubulin gene expression in alcoholics relative to controls. A modest (but non-significant) ≈20% increase of α-tubulin gene expression was observed for suicide alcoholic subjects relative to suicide patients. Beta-tubulin gene expression was also moderately elevated in alcoholic cohorts, but not significantly (Figure 4).

### 3.3. Alcohol Consumption Modelled In Vivo

**Figure 5:**
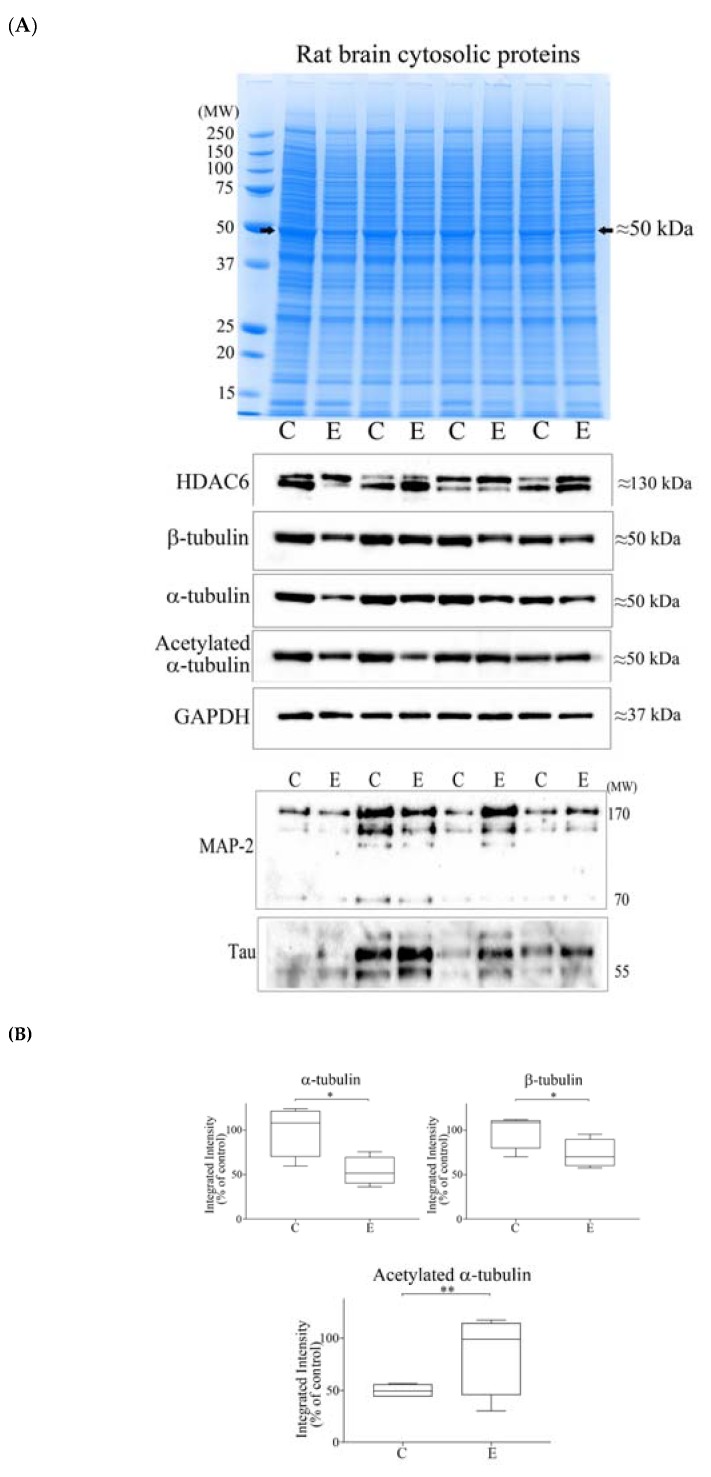
Profiling of brain cytoplasmic proteins from control (C) and ethanol-fed (E) rats, and Western blotting. (**A**) Brain cytosolic proteins from control (C) or ethanol-fed (E) rats resolved by one dimensional polyacrylamide gel electrophoresis were stained with Coomassie (upper panel). Protein staining at ≈50 kDa (marked with an arrowhead) was reduced in ethanol-fed rats. Proteins were Western blotted using a panel of antibodies (middle and lower panels). (**B**) Western blots were quantified by densitometry, with GAPDH levels used for blot normalization, and data presented as box plots. For marked significance: * = *p* < 0.05, ** = *p* < 0.01. Control values were set at 100%.

To examine whether reduced brain tubulin proteins observed for alcoholic subjects (Figure 2 and Reference [10]) could be modelled in vivo, rats were administered alcohol for 4 weeks and then the whole rat brain was homogenized, and proteins were resolved by gel electrophoresis. In keeping with our human *post-mortem* tissue studies (Figure 1), there was reduced protein staining at ≈50 kDa in the brain tissue of alcohol-fed rats (Figure 5A, upper panel). Western blotting confirmed this to be a reduction of α- and β-tubulins and revealed increased α-tubulin acetylation in ethanol-fed rats (Figure 5A, middle panel). However, no significant reductions of HDAC6 (Figure 5A, middle panel), or MAP-2 or MAP-tau proteins (Figure 5A, lower panel) were observed for ethanol-fed rats. Quantitation of Western blots recorded significant 47% (*p* = 0.029) and 38% (*p* = 0.034) reductions of α- and β-tubulins, respectively, and a significant 2.02-fold increase (*p* = 0.003) of α-tubulin acetylation (Figure 5B).

### 3.4. Reductions of α- and β-Tubulin Protein Levels during Ageing

**Figure 6:**
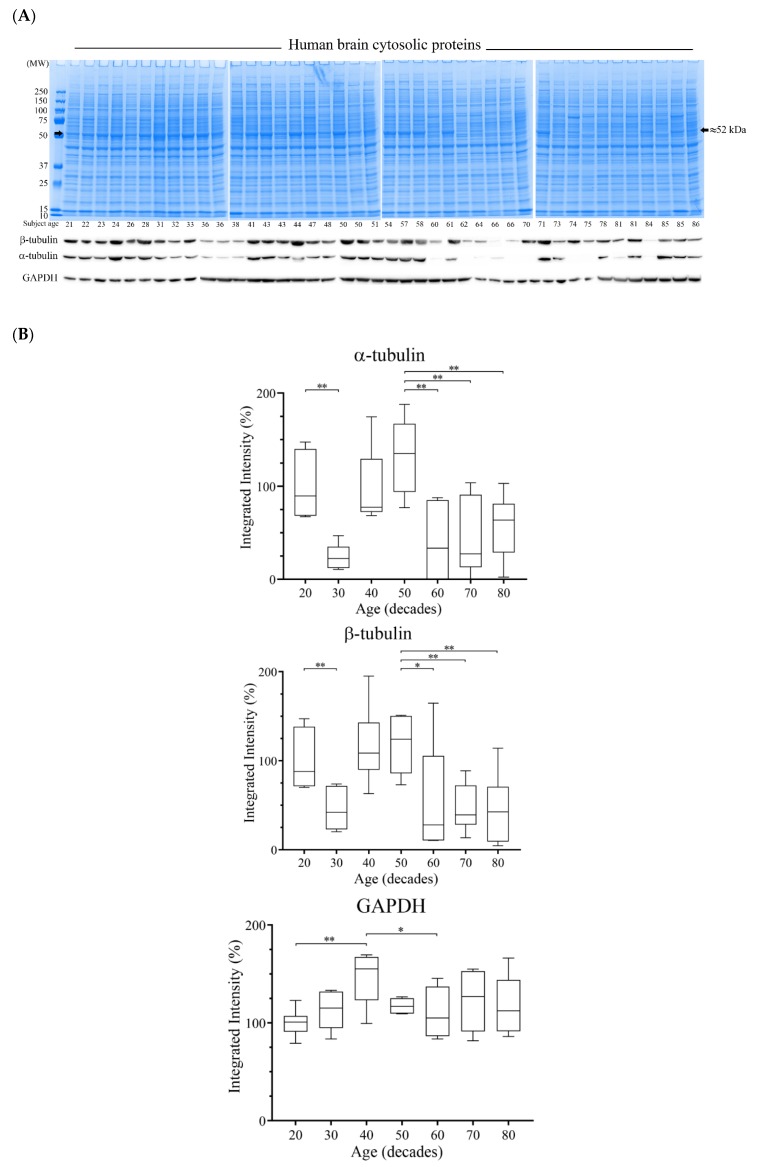

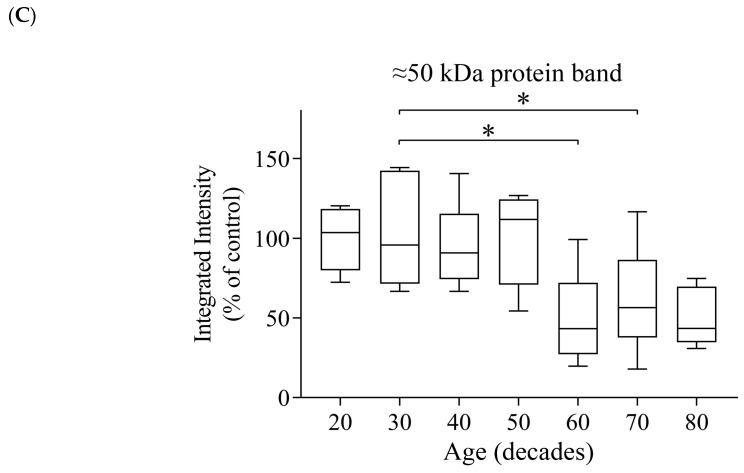
Resolution of proteins from the PFC of humans of progressive age and Western blotting. (**A**) PFC cytosolic proteins were resolved by one dimensional polyacrylamide gel electrophoresis and stained with colloidal Coomassie. A reduction of the protein band at ≈50 kDa was apparent for most subjects over 60 years of age (marked with arrowheads). Western blotting confirmed that the reduced protein staining reflected a lowering of α- and β-tubulin expression. (**B**) Quantification of the changes of protein expression of α- and β-tubulins, and GAPDH across the age range of 21–86 years, with data presented as box plots. GAPDH blots were normalized to total protein staining. For marked significance: * = *p* < 0.05, ** = *p* < 0.01. (**C**) Densitometric quantitation of the ≈50 kDa protein band across the age range of 21–86 years, with data presented as box plots. For marked significance: * = *p* < 0.05.

Neuroimaging studies have detailed a reduction of brain size with subject ageing that is most prominent after middle age [19]. Hence, we also considered whether there was a reduction of tubulin protein expression that was concomitant with normal ageing. Human PFC tissue from control subjects across the age range of 21–86 years (Demographic Table 3) were homogenized and cytosolic proteins were resolved by gel electrophoresis. Protein staining at ≈50 kDa was reduced in the majority of subjects of 60 years of age and above (Figure 6A). Western blotting confirmed that the reduced ≈50 kDa protein staining was attributed to lowered α- and β-tubulin protein expression (Figure 6A).

The relative protein expression patterns of α- and β- tubulins were similar across the ageing time course, with protein expression peaks for subjects aged in their 20s and 50s. Tubulin expression in the 30s was significantly lower than the 20s, and then significantly reduced in the 60s, 70s, and 80s relative to the 50s (Figure 6B). By comparison, GAPDH was stable throughout the lifetime, with a peak in the 40s (Figure 6B). Collectively, there was a negative correlation of tubulin expression with subject age, which was significant for β-tubulin (total subjects as well as female subjects) (Table 4). Interestingly, the dip in α- and β-tubulin levels for subjects in their 30s was not matched by a reduction of protein staining of the ≈50 kDa band (Figure 6C).

## 4. Discussion

Although neuroimaging studies have highlighted brain regional volumetric shrinkage in alcoholic patients [19,20,21,22,23,24,25,26,27], investigations that have addressed whether repeated alcohol exposures induce global neuronal cell death and brain atrophy in chronic alcohol abusers (at autopsy) have been equivocal [11,12,13,14,15,16,17,18]. To provide an insight into alcohol-induced neuronal cell damage, we compared the PFC protein profiles from control, alcoholic, (non-alcoholic) suicide, and suicide alcoholics. In support of our previous studies [10], reduced α- and β-tubulin protein expressions within the cytosol were apparent in alcoholic and suicide alcoholic cohorts. Since the reductions of tubulin protein levels were common to the alcoholic cohorts and not observed with non-alcoholic suicide patients, we suggest that these changes reflect alcohol-induced pathology and not pathology attributed to other psychiatric disorders. That tubulin protein changes were restricted to the cytosol and not evident in either the nuclear or membrane-enriched fractions was consistent with our previous study [10]. Therefore, a further assessment of protein changes in these other subcellular fractions was not conducted.

The tubulin superfamily consists of a broad number of tubulin proteins including α, β, γ, δ, ε, and η forms [40]. The complexity of tubulin expression extends to multiple isoforms, most extensively for α, β, and γ proteins. The formation of cytoplasmic, non-covalent microtubule polymers requires the assembly of α- and β-tubulin heterodimer subunits to form linear, head-to-tail, cylindrical, protofilaments. This formation of microtubules is a dynamic process, with cycles of polymerization and depolymerization [40,41].

In humans, there are multiple, highly homologous isotypes of α- and β-tubulins, including α-tubulin isoforms (α1A, α1B, α1C, α3C, α3D, α3E, α4A, and α8), and β-tubulin isoforms (β, β1, β2a, β2b, β3, β4a, β4b, β6, and β8), encoded by multiple genes that exhibit tissue specific expression [42,43]. Microtubule properties are influenced by which tubulin isoforms assemble, and the site and type of post-translational modifications of the tubulins, including acetylation, that has been collectively termed the “tubulin code” [44,45]. Our mass spectrometry data identified peptides resident to the amino acid sequences of the α1A isoform of α-tubulin, and the β2B isoform of β-tubulin. However, many of these peptides are also present in other highly homologous α- and β-tubulin isotypes, respectively, so precise α- or β-tubulin isotype identification is not feasible. Indeed, a mixed population of α- and β-tubulin isotypes may be present, although identifications of α1A and β2B are consistent with their neuronal gene expressions [42,43].

Collectively, microtubule protofilaments are required for the maintenance of neuronal shape and stability, axonal and neurite growth, and also provide a network for cellular transport activities [40,41]. We would therefore expect that a reduction of tubulin levels as a consequence of chronic alcohol consumption would be refractory to these processes.

Additionally, tubulins provide the scaffolding for a number of associated proteins, and this prompted us to consider whether MAP-2 and MAP-tau were also reduced in alcoholic cohorts. These two MAP-2 family members share a conserved carboxyl-terminal domain containing a number of microtubule-binding repeats [46]. They differ in their cellular localizations with MAP-2 expressed in neuronal dendrites, and MAP-tau predominantly expressed in neuronal axons. Both MAPs exist as multiple protein isoforms derived from alternative splice variants, and both proteins are also subjected to extensive protein post-translational modifications (PTMs) [46,47]. In humans, MAP-2 exists as four main isoforms spanning 70–280 kDa, and these are extensively phosphorylated, producing a complex picture of protein bands after Western blotting (Figure 2B). Likewise, MAP-tau exists as six alternative splice variants, predominantly producing proteins of 50–65 kDa in length [46,48] (Figure 2B). Although prominent reductions were apparent with certain individuals, collectively, significant reductions were not observed for the MAPs when immune detection was averaged across the full cohort. Other published studies also highlight a heterogeneous response of the brain MAP-2 to ethanol. There was a regional reduction of MAP-2 mRNA expression in response to alcohol consumption corrected after alcohol withdrawal [34], and an enhanced MAP-2 protein expression at 50 mM, no change at 100 mM, and then reduced expression at 200 mM in brain slices exposed to ethanol in vitro [49]. Other studies have also reported an ethanol-induced reduction of MAP-2 protein expression after chronic ethanol administration to rats [50], and after alcohol exposure during development of primary neurons [51].

We predict that alcoholic subjects that exhibited a reduced expression of MAP-2 or MAP-tau in conjunction with disrupted tubulin function may display more severe microtubular defects compared to those with tubulin decreases only. However, although knockout of either MAP-2 or MAP-tau results in a reduction of microtubule density in dendrites and axons, respectively, neither knockout produces major deficits in brain morphology, presumably due to redundancy and/or compensatory activity of other proteins or family members [52,53].

We detected increased α-tubulin acetylation and decreased HDAC6 protein levels in the PFC of alcoholic subjects. The regulation of protein acetylation is controlled by the dynamic actions of histone acetyl transferases (HATs) and histone deacetylases (HDACs). Increased α-tubulin acetylation in alcoholic subjects may reflect increased HAT activity, and/or reduced HDAC activity. HDAC6 is the HDAC family member that is able to deacetylate target lysine residues on α-tubulin, and is also able to deacetylate free tubulin dimers and to a lesser extent assembled microtubules [54,55,56]. Presumably, the reduction of HDAC6 protein levels in alcoholic cohorts contributed to the increased α-tubulin acetylation.

The molecular and cellular consequences of increased α-tubulin acetylation in neurons have not been extensively studied. Studies have been restricted to an in vitro demonstration that brain tubulin is sensitive to acetaldehyde adduction, with a corresponding reduction of microtubule stability [57]. Perhaps increased acetylation of neuronal microtubules in vivo increases microtubule stability, but this will need to be determined. Reduced HDAC6 protein levels in alcoholic subjects may also disrupt other cellular processes since HDAC6 deacetylates additional target proteins including Hsp90 and contactin, and also influences autophagic protein degradation and aggresome formation [58]. These autophagic functions provide a means to regulate quality control of proteins, a disturbance of which is linked to a number of neurodegenerative diseases [59].

However, there is a limitation with these analyses in that we and others determine the relative level of α-tubulin acetylation to that of total immune-reactive α-tubulin, and without a determination of absolute acetylation stoichiometry. Hence, there is also the possibility that alcohol-induced PTMs on α-tubulin hinder binding of the full-length α-tubulin antibody, thereby skewing the relative ratio of acetylated to total α-tubulin protein, although clear reductions of tubulin proteins were evident in Coomassie stained gels (Figure 1 and Figure 5).

The gene expressions of α- and β-tubulins were moderately, but not significantly, increased in alcoholic cohorts (Figure 4), possibly to compensate for reduced protein levels. Hence, the relatively low tubulin protein levels detected in alcoholic cohorts was not a consequence of reduced gene transcription. Instead, this reflects a translational or post-translational disturbance, such as increased degradation.

Similar to our human studies, administration of alcohol to rats also resulted in reduced tubulin protein levels. Due to limited sample availability, we examined whole rat brains rather than specific PFC regions. The global effect of alcohol in depleting tubulin in the rat brain was not surprising since we have reported that such defects also arose in brain regions other than the PFC [10]. No changes to MAP-2 or MAP-tau protein levels were recorded after alcohol consumption in rats. Likewise, HDAC6 protein levels were unaltered even though increased α-tubulin acetylation was observed. This may reflect limitations of directly comparing immune-reactive total protein to acetylated protein (*vide supra*), or an alcohol-induced increase of HAT activity or reduced HDAC6 activity.

A major *caveat* to protein studies of *post-mortem* tissue is that it only provides a snap-shot of brain damage at death. Although subjects are matched for age, sex, and tissue *post-mortem* delay, one can only approximate lifetime alcohol consumption, periods of abstinence, and other dietary and lifestyle variables. Nevertheless, loss of tubulin protein from the PFC was evident for the majority of the 22 human alcoholic subjects studied herein, the 20 subjects previously assessed, and for other brain regions examined (caudate nucleus, hippocampus, and cerebellum) [10]. Furthermore, we show that tubulin reductions were observed in our rat model of 4 weeks of alcohol administration, indicating a robust pathological hallmark. In support of our findings, other independent studies have also detected a reduction of α- and β-tubulin levels in human alcoholics [60], reduced tubulin levels in primary neurons [50], and an ethanol-induced disruption of microtubule function and polymerisation [61].

At present we can only speculate as to why tubulin proteins are vulnerable to reduced expression after chronic alcohol consumption. Due to their cellular prevalence, they may be particularly susceptible to protein damage, such as adduction by reactive aldehydes [57], or accumulate isoaspartate protein damage [62,63] that promotes their degradation, but this has not yet been investigated further.

We propose that reduced tubulin proteins could in part explain lowered cortical thickness recorded by neuroimaging studies of chronic alcohol users [64]. Neuroimaging studies have demonstrated that the PFC is particularly susceptible to damage and reduced cortical thickness [12,15,16,17,18,19,20,21,22,23,24,25,26,27,28,29], in keeping with our demonstration that the PFC had the most significant reductions of tubulins when compared to other brain regions examined [10].

We also show that human PFC α- and β-tubulin protein levels were reduced in most subjects between 60 and 86 years of age. Since microtubules are ubiquitous within neurons and extend from cell bodies to promote dendritic arborization, a substantive lowering of tubulin levels is a molecular change able to account for the reduced cortical thickness associated with advancing age [19,28,65,66,67,68,69]. Furthermore, heavy drinking accelerated age-induced brain volumetric deficits [19,20,28,70]. In this study, and in our previous work [10], we have matched our patients for both age and sex, hence tubulin deficits arising from ageing alone were normalized for each control and alcoholic-matched pair. Hence, the additional tubulin deficits observed for alcoholic cohorts must relate to chronic alcohol exposure, and this would be in keeping with neuroimaging studies that suggest that long-term alcohol consumption accelerates volumetric losses. However, in the absence of neuroimaging data for our study participants prior to death, we cannot quantify whether tubulin loss directly correlates with volumetric deficits, or indeed, if tubulin loss is reversible after periods of abstinence. Further in vivo studies using our rat model of organ injury may help to answer these questions.

Across the ageing time course, a reduction of tubulin levels was evident in both male and female subjects, but more pronounced for females. Certainly, there are brain regional heterogenic volume deficits that arise from age [65] and sex [68], but a clearer relationship to both specifically for Brodmann’s area 9 will require increased study numbers.

Noteworthy was the significant reduction of both α- and β-tubulin expression for subjects in their 30s. Although a limitation of our study is the number of data points (six subjects for each decade), four of six subjects from this grouping (30s) had relatively low β-tubulin levels, and all six subjects had relatively low α-tubulin levels, indicative of a consistent affect. Although many of the subjects in their 60s, 70s, or 80s had reduced tubulin levels evidenced via immuno-blotting, this was matched with lowered protein staining at the ≈50 kDa protein region that was comprised of α- and β-tubulin proteins (and other proteins of similar denatured molecular weight) (Figure 6A,B). By contrast, protein staining at the ≈50 kDa region was not significantly reduced for subjects in their 30s (Figure 6C). One possibility to explain this anomaly is that an alteration of α- and β-tubulin isotype expression and/or post-translational modification limits antibody binding, and without a change of tubulin levels. With the production and validation of tubulin isotype-specific antibodies, an analysis of isotype expression across the ageing time course could be conducted to investigate this further.

In contrast to the tubulin protein expression that declines with ageing, we show that GAPDH levels are relatively stable after age 60. GAPDH has a well-documented metabolic role as a glycolytic enzyme, but the GAPDH gene also has a number of associated pseudogenes, and codes for a moonlighting protein with multiple (extra-glycolytic) biological roles [71]. The abundance and assumed stability of GAPDH has promoted its usage as a loading control for Western blotting. Our results (Figure 6), and those of previous studies [10] demonstrate that GAPDH is a suitable reference protein for blotting studies using human brain PFC, caudate nucleus, hippocampus, and cerebellum. Our ageing brain studies also considered total lane protein as a means for blotting normalization [72], and similar declines of tubulin levels due to age or alcohol were evident (results not included).

Interestingly, there was a progressive increase of GAPDH levels from subjects in their 20s to those in their 40s, before a reduction in level for subjects in their 50s, and then stable levels from 60s to 80s. This flattened bell-shape curve for GAPDH levels resembles total white matter brain volume changes with subject ageing reported using magnetic resonance imaging [73], but an over-emphasis on this observation would be unwarranted based on study numbers of six per decade.

## 5. Conclusions

To conclude, tubulin protein levels were dramatically reduced in the PFC as a consequence of chronic alcohol consumption and were also reduced in the majority of subjects from their sixth decade of life. It is possible that tubulin loss could contribute to the reduced brain volume changes evidenced in both alcoholic subjects and the elderly. Furthermore, tubulin loss and damage to the cytoskeletal architecture, or a loss of neurite outgrowth and/or dendritic connections, processes that require functional tubulin proteins, could also explain brain shrinkage in the absence of substantive neuronal cell death and brain atrophy. Structural changes to, and altered connections between, neuronal cells as a consequence of depleted tubulins may contribute to brain functional deficits, and therefore have important implications for the neurobiology and neuropathology of ageing and alcoholism.

## Figures and Tables

**Table 1:** Demographic characteristics, *post-mortem* delay (PMD), psychiatric diagnosis, cause of death, and toxicological characteristics of control (C), alcoholic (A), (non-alcoholic) suicide (S), and suicide alcoholic (SA) subjects.

Case	Gender (F/M)	Age (Years)	PMD (h)	Psychiatric Diagnose	Etiology of Death	Cause of Death	Ethanol in Blood (mg/mL)	Other Drugs in Blood
C1	F	66	15	Control	Accidental	Run over	0	(−)
A1	F	51	8	Alcoholism	Natural	CRF	2.98	Nordiazepam
S1	F	58	48	Major Depression	Suicidal	Drowning	0	(−)
SA1	F	57	24	Alcoholism	Suicidal	Drug intoxication	0.23	Methanol
C2	M	71	19	Control	Natural	CRF	0	Nordiazepam
A2	M	68	15	Alcoholism	Natural	CRF	0	(−)
S2	M	65	19	Obsessive Disorder	Suicidal	Jumping	0	Nordiazepam
SA2	M	71	19	Alcoholism	Suicidal	Hanging	0.55	(−)
C3	M	48	7	Control	Accidental	Traffic	0	(−)
A3	M	50	24	Alcoholism	Natural	CRF	0	(−)
S3	M	50	6	Bipolar Disorder	Suicidal	Jumping	0	Diazepam
SA3	M	50	3	Alcoholism	Suicidal	Hanging	0	(−)
C4	M	52	18	Control	Accidental	Crushed	0	(−)
A4	M	57	12	Alcoholism	Natural	CRF	3.37	(−)
S4	M	59	29	Major Depression	Suicidal	Hanging	0	Citalopram, Nordiazepam
SA4	M	55	24	Alcoholism	Suicidal	Drug intoxication	2.4	(−)
C5	M	40	18	Control	Accidental	Jumping	0.56	(−)
A5	M	43	4	Alcoholism	Natural	CRF	1.64	Chlormethiazole, Metamizole
S5	M	44	15	Anxiety Disorder	Suicidal	Hanging	0	(−)
SA5	M	40	21	Alcoholism	Suicidal	Run over	0	(−)
C6	F	36	9	Control	Natural	Heart failure	0	(−)
A6	F	43	35	Alcoholism	Natural	Hemorrhage	0	Metamizole, Fluoxetine
S6	F	48	9	Anxiety Disorder	Suicidal	Jumping	0	(−)
SA6	F	50	6	Alcoholism	Suicidal	Jumping	0	Hydrochlorothiazide
C7	M	66	50	Control	Accidental	Traffic	0	(−)
A7	M	71	17	Alcoholism	Accidental	Traffic	0.35	(−)
S7	M	67	8	Major Depression	Suicidal	Sharp weapon	0	(−)
SA7	M	69	24	Alcoholism	Suicidal	Jumping	0.38	(−)
C8	M	37	21	Control	Accidental	Cranoencephalic trauma	0	(−)
A8	M	39	19	Alcoholism	Natural	Hemorrhage	0.44	(−)
S8	M	40	15	Personality Disorder	Suicidal	Jumping	0	Diazepam
SA8	M	37	68	Alcoholism	Suicidal	Hanging	0.9	Diazepam
C9	M	54	23	Control	Accidental	Jumping	0	(−)
A9	M	53	12	Alcoholism	Natural	Suffocation	0	(−)
S9	M	50	29	Major Depression	Suicidal	Hanging	0	Diazepam
SA9	M	50	70	Alcoholism	Suicidal	Hanging	2.93	(−)
C10	M	42	27	Control	Accidental	Traffic	0	(−)
A10	M	42	20	Alcoholism	Natural	Hemorrhage	0	(−)
S10	M	41	50	Major Depression	Suicidal	Hanging	0	(−)
SA10	M	41	78	Alcoholism	Suicidal	Hanging	3.4	(−)
C11	M	47	26	Control	Accidental	Work accident	0	(−)
A11	M	46	16	Alcoholism	Accidental	Suffocation	0.97	(−)
S11	M	46	47	Anxiety Disorder	Suicidal	Hanging	0	(−)
SA11	M	44	5	Alcoholism	Suicidal	Jumping	2.47	Diazepam

F, Female; M, male; CRF, cardio-respiratory failure

**Table 2:** Means and gender distribution of the demographic characteristics of the subjects used.

	Controls	Alcoholic Subjects	Suicide Subjects	Suicide Alcoholic Subjects
**Age (years)**	50 ± 4	51 ± 3	52 ± 3	51 ± 3
**PMD (h)**	21 ± 3	17 ± 3	25 ± 5	31 ± 8
**Gender (F/M)**	2F/9M	2F/9M	2F/9M	2F/9M

**Table 3:** Demographic characteristics, *post-mortem* delay (PMD), and cause of death of the control subjects used for the ageing study.

Case	Gender (F/M)	Age (Years)	PMD (h)	Cause of Death
1	M	21	15	Accident/Traffic
2	F	22	24	Accident/Fall from height
3	M	23	17	Accident/Work
4	M	24	20	Accident/Traffic
5	F	26	5	Accident/Traffic
6	M	28	5	Accident/Fall from height
7	M	31	13	Accident/Traffic
8	M	32	27	Accident/Traffic
9	M	33	13	Accident/Traffic
10	M	36	18	Accident/Work
11	M	36	23	Accident/Work
12	F	38	22	Accident/Traffic
13	M	41	14	Natural/Heart Attack
14	M	43	10	Accident/Traffic
15	F	43	28	Accident/Train
16	F	44	9	Natural/CRF
17	M	47	15	Accident/Traffic
18	F	48	9	Accident/Fall from height
19	F	50	11	Natural/CRF
20	F	50	11	Natural/CRF
21	M	51	18	Accident/Traffic
22	M	54	24	Accident/Traffic
23	F	57	14	Natural/CRF
24	M	58	16	Accident/Traffic
25	F	60	27	Accident/Traffic
26	M	61	23	Accident/Traffic
27	M	62	19	Accident/Work
28	F	64	19	Natural/CRF
29	F	66	20	Natural/CRF
30	M	66	18	Natural/Tumor
31	F	70	7	Accident/Traffic
32	M	71	21	Natural/CRF
33	M	73	19	Accident/Traffic
34	F	74	21	Accident/Traffic
35	F	75	18	Accident/Traffic
36	M	78	29	Accident/Traffic
37	F	81	14	Accident/Traffic
38	M	81	21	Accident/Traffic
39	F	84	18	Natural/CRF
40	M	85	19	Natural/CRF
41	M	85	10	Natural/CRF
42	F	86	18	Natural/CRF

F, Female; M, male; CRF, cardio-respiratory failure

**Table 4:** Correlation between protein expression level, and sex and age of subjects.

	α-Tubulin	β-Tubulin	GAPDH
**All subjects**	42	42	42
Spearman’s Correlation Coefficient	−0.225	−0.350 *	0.068
Significance (two-tailed)	0.153	0.023	0.67
**Male Subjects**	24	24	24
Spearman’s Correlation Coefficient	−0.107	−0.247	0.303
Significance (two-tailed)	0.617	0.244	0.15
**Female Subjects**	18	18	18
Spearman’s Correlation Coefficient	−0.392	−0.510 *	−0.274
Significance (two-tailed)	0.107	0.031	0.272

Footnote: * = correlation significant with *p* < 0.05.

## Supplementary Materials

The following are available online at http://www.mdpi.com/2076-3425/8/9/175/s1.
