# Supplementary material for: The Loss of α- and β-Tubulin Proteins Are a Pathological Hallmark of Chronic Alcohol Consumption and Natural Brain Ageing
Source: Brain Sci. 2018 Sep 11;8(9):175. doi: 10.3390/brainsci8090175 (PMC6162390; doi:10.3390/brainsci8090175)
Supplement: Supplementary file 1 [file brainsci-08-00175-s001.pdf]

Supplementary Data:

Mass spectrometric analysis was performed on the ~50 kDa protein band from control, suicide, alcoholic, or suicide alcoholic subjects. Matrix-assisted laser desorption ionization-time of flight (MALDI-TOF) mass spectrometry identified twelve tryptic peptides that constituted ~42% coverage of tubulin subtype α1A. These peptides generated a MOWSE probability score of 117.

The ~50 kDa protein band also liberated fifteen peptides that corresponded to approximately 45 % protein coverage of β-2B-tubulin, and generated a MOWSE probability score of 159. For this MALDI-TOF mass spectrometry analysis a MOWSE score of greater than 66 was considered a significant match ($P < 0.05$) of tryptic peptides to those present within protein databases, to enable confident protein identification.

Tubulin α-1A-chain peptides:

St-End	Observed	Mr(expt)	Mr(calc)	Delta	Sequence
85-96	1410.8100	1409.8027	1409.7667	0.0360	R.**QLFHPEQLITGK**.E
97-105	1023.4700	1022.4627	1022.4417	0.0210	K.**EDAANNYAR**.G
113-121	1069.6200	1068.6127	1068.5815	0.0312	K.**EIIDPVLDR**.I
216-229	1718.8800	1717.8727	1717.8747	-0.0020	R.**NLDIERPTYTNLNR**.L
230-243	1487.9000	1486.8927	1486.8719	0.0209	R.**LISQIVSSITASLR**.F
244-264	2409.1900	2408.1827	2408.2012	-0.0185	R.**FDGALNVDLTEFQTNLVPYPR**.I
265-280	1756.9500	1755.9427	1755.9559	-0.0132	R.**IHFPLATYAPVISAEK**.A
281-304	2750.4100	2749.4027	2749.2840	0.1188	K.**AYHEQLSVAEITNACFEPANQMVK**.C
312-320	1249.5700	1248.5627	1248.5453	0.0174	K.**YMACCLLYR**.G
353-370	1824.9300	1823.9227	1823.9782	-0.0554	K.**VGINYQPPTVVPGGDLAK**.V
374-390	1864.8800	1863.8727	1863.8971	-0.0244	R.**AVCMLSNTTAIAEAWAR**.L
403-422	2330.0200	2329.0127	2329.0110	0.0018	R.**AFVHWYVGEGMEEGEFSEAR**.E

The positions of the tryptic peptides detected within the human α-1A-tubulin amino acid sequence are also shown in bold. The position of K-40, the site of acetylation recognised by a mouse monoclonal antibody is underlined.

1 MRECISIHVG QAGVQIGNAC WELYCLEHGI QPDGQMPSDK TIGGGDDSFN
51 TFFSETGAGK HVPRAVFVDL EPTVIDEVRT GTYR**QLFHPE** **QLITGKEDAA**
101 **NNYAR**GHYTI GK**EIIDLVLD** **R**IRKLADQCT GLQGFLVFHS FGGGTGSGFT
151 SLLMERLSVD YGKKSKLEFS IYPAPQVSTA VVEPYNSILT THTTLEHSDC
201 AFMVDNEAIY DICRR**NLDIE** **RPTYTNLNRL** **IGQIVSSITA** **SLRFDGALNV**
251 **DLTEFQTNLV** **PYPRIHFPLA** **TYAPVISAEK** **AYHEQLSVAE** **ITNACFEPAN**
301 **QMVK**CDPRHG K**YMACCLLYR** GDVVPKDVNA AIATIKTKRT IQFVDWCPTG
351 FK**VGINYQPP** **TVVPGGDLAK** VQR**AVCMLSN** **TTAIAEAWAR** LDHKFDLMYA
401 KR**AFVHWYVG** **EGMEEGEFSE** **AR**EDMAALEK DYEEVGVDSV EGEGEEEGEE
451 Y

Tubulin β-2B-chain peptides:

St-End	Observed	Mr(expt)	Mr(calc)	Delta	Sequence
3-19	1822.9000	1821.8927	1821.9156	-0.0228	R.**EIVHIQAGQCGNQIGAK**.F
47-58	1355.6800	1354.6727	1354.6517	0.0210	R.**INVYYNEAAGNK**.Y
63-77	1615.8500	1614.8427	1614.8287	0.0140	R.**AILVDLEPGTMDSVR**.S
78-103	2798.5400	2797.5327	2797.3361	0.1966	R.**SGPFGQIFRPDNFVFGQSGAGNNWAK**.G
104-121	1958.9300	1957.9227	1957.9745	-0.0518	K.**GHYTEGAELVDSVLDVVR**.K
155-162	1077.5500	1076.5427	1076.5250	0.0177	K.**IREEYPDR**.I
157-174	2141.0000	2139.9927	2139.9969	-0.0042	R.**EEYPDRIMNTFSVMPSPK**.V
217-241	2708.4000	2707.3927	2707.3310	0.0618	K.**LTTPTYGDLNHLVSATMSGVTTCLR**.F
242-251	1130.6300	1129.6227	1129.5880	0.0347	R.**FPGQLNADLR**.K
253-262	1143.6500	1142.6427	1142.6270	0.0157	K.**LAVNMVPFPR**.L
263-276	1620.8600	1619.8527	1619.8283	0.0245	R.**LHFFMPGFAPLTSR**.G
298-306	1065.4700	1064.4627	1064.4201	0.0426	K.**NMMAACDPR**.H
310-318	1053.6200	1052.6127	1052.6019	0.0109	R.**YLTVAAIFR**.G
351-359	1028.5500	1027.5427	1027.5121	0.0307	K.**TAVCDIPPR**.G
381-390	1229.6200	1228.6127	1228.5910	0.0217	R.**ISEQFTAMFR**.R

The positions of the tryptic peptides detected within the human β-2B-tubulin amino acid sequence are also shown in bold:

1 MR**EIVHIQAG QCGNQIGAK**F WEVISDEHGI DPTGSYHGDS DLQLER**INVY**
51 **YNEAAGNK**YV PR**AILVDLEP GTMDSVRSGP FGQIFRPDNF VFGQSGAGNN**
101 **WAKGHYTEGA ELVDSVLDVV R**KESESCDCL QGFQLTHSLG GGTGSGMGTL
151 LISK**IREEYP DRIMNTFSVM PSPK**VSDTVV EPYNATLSVH QLVENTDETY
201 SIDNEALYDI CFRTLK**LTTP TYGDLNHLVS ATMSGVTTCL RFPGQLNADL**
251 **R**K**LAVNMVPF PRLHFFMPGF APLTSR**GSQQ YRALTVPELT QQMFDSK**NMM**
301 **AACDPR**HGR**Y LTVAAIFR**GR MSMKEVDEQM LNVQNKNSSY FVEWIPNNVK
351 **TAVCDIPPR**G LKMSATFIGN STAIQELFKR **ISEQFTAMFR** RKAFLHWYTG
401 EGMDEMEFTE AESNMNDLVS EYQQYQDATA DEQGEFEEEE GEDEA
